# A Complicated Case of Postpartum Myasthenic Crisis

**DOI:** 10.7759/cureus.20247

**Published:** 2021-12-07

**Authors:** Ryan Quigley, Zachary A Koenig, Samuel Schick, Erin Miller

**Affiliations:** 1 Department of Medicine, West Virginia University, Morgantown, USA; 2 Department of Obstetrics and Gynecology, West Virginia University, Martinsburg, USA

**Keywords:** perforated appendicitis, myasthenia gravis, covid-19, myasthenic crisis, postpartum

## Abstract

Myasthenia gravis (MG) is the most common autoimmune disorder affecting the neuromuscular junction (NMJ) of voluntary skeletal muscle. This disease is characterized by ptosis, diplopia, facial muscle weakness, bulbar muscle involvement including dysphagia and difficulty chewing, dysarthria, hypophonia, respiratory muscle fatigue, and sometimes generalized weakness. A myasthenic crisis (MC) is a complication of MG. MC is defined as severe worsening of respiratory function necessitating the need for mechanical ventilation. Precipitating factors include infection, certain drugs, pregnancy, childbirth, surgery, discontinuation of medical therapy, or even spontaneously with no inciting event. Here we present a complicated case of a 24-year-old patient with a long history of controlled who encounters many events that lead to an MC necessitating mechanical intubation, plasmapheresis, and high dose immunosuppressive therapy. She recently gave birth to a child, had an occult perforated appendicitis with multiple abscesses needing emergent exploratory laparotomy, and had an overlying COVID-19 infection. The complexity of this disease and its complications warrants careful consideration by physicians in any branch of specialty.

## Introduction

Myasthenia gravis (MG) is the most common autoimmune disorder affecting the neuromuscular junction (NMJ) of voluntary skeletal muscle. Prevalence ranges from 14 to 20 per 100,000 people with an estimated 50,000 cases in the United States. One in 20,000 pregnant women has this condition [1]. It involves the formation of autoantibodies originating in the thymus to post-synaptic receptors of the NMJ including the nicotinic acetylcholine receptors (AChR), muscle-specific tyrosine kinase (MuSK), a combination of the two, or to neither one making these types of patients seronegative which account for 6% to 12% of MG patients [2]. Other less frequently clinically tested antibodies include anti-LRP4, agrin, and titin proteins [3]. Autoantibodies to the AChR competitively inhibit the binding of acetylcholine causing receptor decay through internalization and activation of the complement system leading to impaired signal transduction causing skeletal muscle weakness and fatigue. Antibodies to MuSK reduce clustering of the proteins needed to stabilize the AChR on the post-synaptic membrane [4]. Other associated autoimmune conditions that share similar human leukocyte antigens predisposing subtypes include Hashimoto thyroiditis, Graves disease, rheumatoid arthritis, sarcoidosis, Addison disease, and systemic lupus erythematosus.

This disease is characterized by ptosis, diplopia, facial muscle weakness, bulbar muscle involvement including dysphagia and difficulty chewing, dysarthria, hypophonia, respiratory muscle fatigue, and sometimes generalized weakness. These symptoms classically worsen with repetitive muscle use as acetylcholine is degraded by the enzyme acetylcholine esterase (AChE), making acetylcholine less likely to interact with the limited number of available AChR. Treatment is focused on improving symptoms by inhibiting AChE with drugs such as pyridostigmine to increase the synaptic concentration of acetylcholine. If symptoms persist despite this treatment, additional immunosuppressive therapy targeting the immune dysregulation of their disease is warranted with drugs such as glucocorticoids, azathioprine, rituximab, methotrexate, cyclophosphamide, cyclosporine, and mycophenolate mofetil. Prognosis is very a good given treatment. Most poorly treated patients die from exacerbations of symptoms leading to respiratory failure.

Up to 50% of patients have an associated thymoma that paraneoplastically manifests as MG. Patients diagnosed with MG typically undergo mediastinal imaging to identify the presence of an anterior mediastinal mass. These can be surgically resected for the dual purpose of tumor removal and management of MG disease. If no mass is found, patients can be stratified into two groups to determine if thymectomy is warranted due to evidence showing resection on non-diseased thymus improves symptoms. Patients less than 60 years old and have AChR positive antibodies or are seronegative benefit from thymectomy whereas patients older than 60 or are MuSK antibody-positive show no benefit from thymectomy [5]. Interestingly, MG patients without a thymoma who undergo thymectomy respond better than MG patients with a thymoma [6].

Pregnant women with MG usually experience an exacerbation of their symptoms in the first month following delivery or during the first trimester of pregnancy with improvement during the remainder of the pregnancy [7]. Exacerbations stem from respiratory muscle fatigue from hypoventilation due to elevation of the diaphragm from a gravid uterus, infections during pregnancy, as well as the stress of labor and delivery [8]. Some studies show the greatest risk of mortality during pregnancy correlates positively with the number of years from disease onset with the highest risk one year after symptoms manifest [9].

Pyridostigmine and immunosuppressive therapy should continue throughout pregnancy to control the mothers' symptoms and reduce the risk of transient neonatal myasthenia and the very rare disease arthrogryposis multiplex congenita, which is characterized by congenital joint contraction and muscle weakness of the limbs due to inhibition of normal joint movement before birth, lung hypoplasia, and perinatal death. Overall, MG does not affect the development of the child. There is an increased risk of premature rupture of membranes in the case of congenital myasthenia in the setting of polyhydramnios caused by the inability of fetal swallowing mechanisms [10]. Mycophenolate, azathioprine, methotrexate, cyclophosphamide have known teratogenic effects and should not be used during pregnancy. Rituximab has shown no major adverse effects on the fetus, but studies are limited. Its use for severe MG and MuSK positive MG should make its use limited to cases where the benefit to the mother clearly outweighs the possible risk to the fetus. Frequent ultrasounds of the fetus should be used in these cases [10]. Thymectomy is also recommended before becoming pregnant while considering that there is some lag time for the effects of this surgery to become apparent. However, thymectomy should not occur during pregnancy [11].

A myasthenic crisis (MC) is a complication of MG experienced by 20% of patients. MC is defined as severe worsening of respiratory function necessitating the need for mechanical ventilation. Mortality rate ranges from 5% to 12% [12]. Severe bulbar muscle weakness may be the most predominant warning sign in these patients. Precipitating factors include infection, pregnancy, childbirth, surgery, discontinuation of medical therapy, or even spontaneously with no inciting event. Certain drugs are known to exacerbate MG by interfering with NMJ transmission including antibiotics such as aminoglycosides and fluoroquinolones, beta-blockers, and magnesium to name a few. MG is also very sensitive to anesthetics targeting the NMJ. Patients often need increased doses of succinylcholine and decrease doses of nondepolarizing neuromuscular blockers [12]. Treatment of an MC crisis includes early mechanical intubation when respiratory failure seems inevitable. Signs to note are a negative inspiratory force (NIF) below 30 cm H_2_O, a forced vital capacity (FVC) below 20mL/kg, clinical signs of respiratory distress, or evidence of respiratory acidosis. Pyridostigmine should be discontinued in intubated patients to avoid airway over secretions by the parasympathetic nervous system [12] Other therapy includes the use of intravenous immunoglobulins (IVIg) daily for five days or plasmapheresis five times over the course of seven to 14 days. High-dose immunosuppressive therapy is also warranted. Treatment in a pregnant patient does not change. Avoidance of teratogenic immunosuppressives, careful monitoring of a hypovolemic state during plasmapheresis to insure adequate placental perfusion, and monitoring for hyperviscosity syndromes during IVIg therapy should all be considered [8].

## Case presentation

We present a case of a 24-year-old G3P2012 with a past medical history of MG status post thymectomy in 2008 currently controlled with pyridostigmine 60mg four times daily. She underwent a successful medically indicated induction of labor at 38 weeks one day for pregnancy complicated with fetal growth restriction. She was compliant with her prenatal care. During admission for labor, she was found to have a COVID-19 infection with symptoms of sinus congestion for a couple of days but no fever. Her induction was uncomplicated and resulted in the delivery of a live female neonate, weighing 2.26 kg with Apgar scores of 9 and 9 at one and five minutes, respectively. While hospitalized, she received aspirin, vitamin C, vitamin D, and zinc due to her COVID-19 positive status. Of note, after her prior delivery, she required an increase in her dose of pyridostigmine due to MC. Therefore, she was observed until postpartum day 3 and was found to be recovering appropriately without signs or symptoms of an MC. She was discharged home in stable condition.

**Figure 1:**
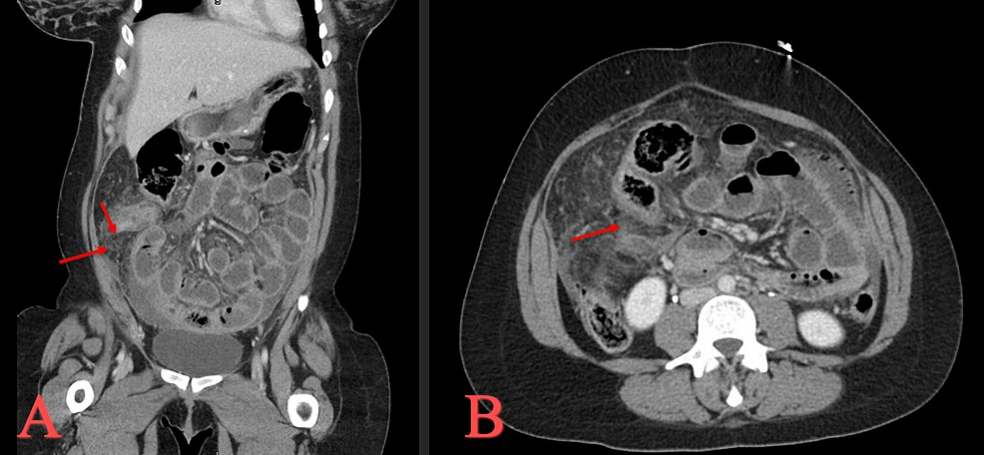
CT of the abdomen and pelvis during the initial presentation. (A) Coronal view with red arrows to demonstrate extensive inflammatory changes found around the appendix. (B) Axial view with red arrow to demonstrate that the appendix is thickened and the walls are not well defined, suggesting ruptured appendicitis.

Four days after discharge, she presented to the emergency department with a chief complaint of worsening generalized abdominal pain and shortness of breath since the delivery of her baby. She had stable vital signs. Physical exam revealed a distended abdomen with severe tenderness to palpation in all four quadrants. CT of the abdomen and pelvis with IV contrast showed marked inflammatory changes in the right lower quadrant, multiple interloop abscesses, and a ruptured appendix (Figures 1A, 1B). There was fluid in the mid-right flank and in Morrison’s pouch. Labs showed a WBC of 29.9 with neutrophilic predominance. The patient was taken for emergent exploratory laparotomy.

Upon entry into the abdomen, there was foul-smelling discharge between the omentum and parietal peritoneum. There was a large phlegmon fusing the omentum to the ascending colon, cecum, terminal ileum, and a few feet of the small bowel. Numerous distended, edematous, and fluid-filled loops of small bowel were seen. Continuous aspiration and irrigation with normal saline and antibiotic fluid of the abdomen occurred. The initial incision was extended due to multiple intra-loop abscesses and areas of purulent fluid secretions. The appendix appeared necrotic and perforated. It was removed with no damage to the cecum or terminal ileum. The entire matted phlegmonic area was also delivered through the wound. Two Jackson-Pratt drains and a Wound VAC were applied. The patient was hemodynamically stable during the procedure, extubated in good condition, and transferred to the intensive care unit for close monitoring.

Throughout the night, the patient experienced persistent sinus tachycardia unresponsive to fluid boluses. Blood pressure was stable. Oxygen saturation was stable on a 6L/min nasal cannula. The potential for an MC was monitored with frequent checks of her NIF, FVC, arterial blood gases, and neurological exam checks. Pyridostigmine was continued. She began experiencing generalized weakness and shortness of breath six hours post-op. There were no difficulties swallowing or coughing. There was no diplopia or ptosis. Over the span of eight hours post-op, her NIF worsened from -40 cm H_2_O to -22 cm H_2_O. After discussion, the patient agreed to rapidly sequence intubation to protect her respiratory function. The patient stated before intubation that her generalized weakness was identical to her prior MG flare-ups. She was also started on 80mg prednisone. The patient reports a history of an anaphylactic reaction to IVIg, so the decision was made to transfer to a tertiary care facility where she could undergo plasmapheresis for the suspected MC.

On arrival, another dose of 62.5 mg of solumedrol was given. She was then started on dexamethasone 6mg daily. She had a fever of 102.9. Improved WBC of 16.5. ESR 42. Procalcitonin 2.29. CRP 410.6. CXR showed moderate patchy infiltrates bilaterally and a small left-sided pleural effusion indicating an exacerbation of her respiratory status from possible worsening COVID-19 infection. One round of plasmapheresis was made the next day. Two days after intubation, the patient began to improve and was weaned off her ventilator settings. She was successfully extubated on post-op day three. Blood collected prior to plasmapheresis returned AChR and MuSK antibody negative. The patient recovered remarkably and was discharged home six days after initial intubation.

## Discussion

Appendicitis is one of the most common general surgery emergencies encountered in a pregnant woman with an estimated incidence of 1,500 to 2,000 cases per pregnancy [13]. There appears to be an equal distribution of cases in each trimester. Every clinician should have a high index of suspicion for appendicitis in a pregnant woman due to the fact that a perforated appendix is the number one surgical cause of fetal demise [13]. Patient education about the signs and symptoms of appendicitis should be discussed. The most common symptom of appendicitis during pregnancy is periumbilical pain that migrates to the RLQ of the abdomen along with nausea, vomiting, and loss of appetite. It is easy to see how anyone could mistake these symptoms for a normal pregnancy. RLQ pain is also commonly not seen in patients after the fifth month of gestation as the appendix can migrate superiorly above the iliac crest with the tip pointed medially due to the mass effect from a gravid uterus. This lessens the burden of peritoneal irritation due to greater separation of the inflamed appendix from the anterior abdominal wall [13]. A gravid uterus can also interfere with the ability of the omentum to wall off an inflammatory process, leading to a higher incidence of extra-appendiceal infection on presentation. Leukocytosis is also hard to interpret as this can be a normal finding during pregnancy, but pandemia remains a good indicator for suspicion of appendicitis given the correct clinical scenario.

Further investigation with radiological studies is warranted with pregnant patients presenting with symptoms of appendicitis. Ultrasound remains the safest and most reliable method for diagnosing appendicitis while ruling out other causes of abdominal pain including ovarian torsion, pancreatitis, biliary disease, urolithiasis, or the most common misdiagnosis in pregnant patients treated for acute appendicitis, pyelonephritis. If the appendix fails to be visualized with ultrasound, the next step in the evaluation should be MRI. CT scan should only be used for complicated cases or when MRI cannot be utilized at the host institution. As mentioned earlier, the risk of fetal demise due to perforated appendicitis may warrant the necessity of exposing the fetus to harmful radiation. Treatment is immediate surgical intervention [14]. Our patient presented two weeks prior to her emergency surgery with symptoms of RLQ pain. An ultrasound should have been conducted at that time. Failure to do so leads to insufficient medical care that could have prevented the more stressful and lengthy exploratory laparotomy, decreasing the chances of inducing an MC.

If surgery is indicated for patients with MG, many preoperative, operative, and post-operative considerations need to be made. All decisions should be discussed with a multi-disciplinary team. Elective surgery should only be considered if the patient is clinically stable with regard to their MG symptoms. Past research has shown many preoperative factors that increase the chances of developing postoperative MC (POMC) including; vital capacity <2 to 2.9 L, the onset of MG symptoms greater than six years prior, current pyridostigmine dose >750 mg/day, history of pulmonary dysfunction, preoperative bulbar symptoms, prior history of an MC, intraoperative blood loss >1L, and serum AChR antibody >100 nmol/mL [15]. Our patient did not have any of these risk factors besides a greater than a six-year duration of her MG symptoms.

If elective or emergent surgery is considered, many things can be done to lower the chances of developing POMC. Anticholinesterase agents should be continued until the morning of the surgery as discontinuation of these medications can lead to abrupt weakness in some patients who are sensitive. This may lead to a delay in the effect of non-depolarizing muscle relaxers, but some studies suggest that it reduces the risk of respiratory discomfort in most patients [16]. If the patient is taking steroids, evaluation for hypothalamic-pituitary axis suppression should be made and treated accordingly. Patients without signs of hypothalamic-pituitary axis suppression should not receive steroids preoperatively since they are known to initially cause weakness. There is however sufficient evidence of administering IVIg for two to five days or plasmapheresis three to five times over seven to 15 days before undergoing elective surgery. These treatments should be timed to end one week prior to surgery [17]. A previous case report from 2009 demonstrated a successful planned C-section in a patient with MG who underwent plasmapheresis three weeks before her surgery [14]. The patient did not experience any symptoms of an MC. No studies have been conducted on the use of these treatments preoperatively for acute surgical emergencies such as the scenario encountered by our patient.

There are many considerations to be made with regards to the method of anesthesia for patients with MG. Given the pathophysiology of MG, patients will need increased doses of succinylcholine and decreased doses of nondepolarizing neuromuscular blockers. These should be avoided as patients with MG have unpredictable responses to neuromuscular blockers and their reversal agents. Total intravenous anesthesia with propofol and remifentanil have successfully been used during surgery without using neuromuscular blockers [16,17]. If neuromuscular blockers are used, rocuronium or vecuronium should be considered along with the reversal agent sugammadex if available [8,17]. Signs of a cholinergic crisis should be monitored in every patient. If possible, the use of local or regional anesthesia should be used with amide anesthetics [18]. Additionally, nerve blocks that could impair the function of the accessory muscles of respiration or the phrenic nerve such as a mid-thoracic neuraxial or brachial plexus block should be used with caution.

One case report previously showed a successful urgent exploratory laparotomy for small bowel obstruction in the terminal ilium caused by a foreign body on a patient with MG while only utilizing low-dose spinal anesthesia [18]. The spinal anesthesia was introduced at the L2/L3 intervertebral space with bupivacaine and fentanyl. Pinprick test revealed bilateral block at the level of T3. The patient was placed 15 degrees head down to maintain cardiac output while reducing the chance of hypotension during the procedure given the location of the block. Her procedure lasted 1.5h and consisted of an ileectomy with ileocecal anastomosis. Her motor and sensory function recovered two hours after the procedure. She was discharged without suffering any signs of an MC or respiratory distress. She originally presented with ptosis, respiratory dysfunction, mild generalized weakness, and a history of MG for the last eight years with concurrent use of pyridostigmine 60mg every eight hours. She was very high risk for developing an MC, but perhaps their strategy of local spinal anesthesia rather than general anesthesia prevented any adverse events from happening. This method of anesthesia could have been implemented into our patient’s exploratory laparotomy to decrease the use of general anesthesia and its associated poor outcomes in patients with MG.

Given the recent COVID-19 pandemic, it is important to understand its impact on this unique population. Many patients with MG are taking immunosuppressive medications and are therefore at increased risk for worse outcomes if infected by SARS-CoV-2. Furthermore, medications used to treat COVID-19, such as azithromycin, can exacerbate MG symptoms. One study published in The Lancet looked at the rate of MG worsening and the rate of developing an MC in patients infected with SARS-CoV-2 [19]. Thirty-six of the 91 patients included in the study experienced a worsening of their symptoms or developed an MC. Twenty-two patients died due to the infection. This places the mortality rate well beyond that of the general population. Going forward, it is important to consider that this population is in desperate need of early vaccination of any potential respiratory illness.

## Conclusions

This case highlights the importance of being proactive to decrease the chances of developing an MC in patients with MG. Our patient had a diverse set of risk factors for developing an MC that required a multitude of specialties to collaborate and decide the best plan of treatment. Our patient recently gave birth, had an upper respiratory infection caused by SARS-CoV-2, and was surgically treated for perforated appendicitis with multiple intra-abdominal abscesses. We highlight the importance of early ultrasonography in pregnant women presenting with abdominal pain to prevent the worsening of acute appendicitis.
